# Experiences of working as early career allied health professionals and doctors in rural and remote environments: a qualitative systematic review

**DOI:** 10.1186/s12913-022-08261-2

**Published:** 2022-07-26

**Authors:** Alison Dymmott, Stacey George, Narelle Campbell, Chris Brebner

**Affiliations:** 1 Flinders University Caring Futures Institute, College of Nursing and Health Sciences, Flinders University, Adelaide, South Australia Australia; 2 Flinders University Northern Territory, College of Medicine and Public Health, Flinders University, Darwin, Northern Territory Australia

**Keywords:** Allied health, Early career, Experiences, Medicine, Rural and remote, Systematic review, Qualitative, Meta-synthesis

## Abstract

**Background:**

Maintaining a health professional workforce in rural and remote areas poses a significant challenge internationally. A range of recruitment and retention strategies have had varying success and these are  generally developed from the collective experience of all health professions, rather than targeted to professional groups with differing educational and support contexts. This review explores, compares and synthesises the evidence examining the experience of early career rural and remote allied health professionals and doctors to better understand both the profession specific, and common factors that influence their experience.

**Methods:**

Qualitative studies that include early career allied health professionals’ or doctors’ experiences of working in rural or remote areas and the personal and professional factors that impact on this experience were considered. A systematic search was completed across five databases and three grey literature repositories to identify published and unpublished studies. Studies published since 2000 in English were considered. Studies were screened for inclusion and critically appraised by two independent reviewers. Data was extracted and assigned a level of credibility. Data synthesis adhered to the JBI meta-aggregative approach.

**Results:**

Of the 1408 identified articles, 30 papers were eligible for inclusion, with one rated as low in quality and all others moderate or high quality. A total of 23 categories, 334 findings and illustrations were aggregated into three synthesised findings for both professional groups including: making a difference through professional and organisational factors, working in rural areas can offer unique and rewarding opportunities for early career allied health professionals and doctors, and personal and community influences make a difference. A rich dataset was obtained and findings illustrate similarities including the need to consider personal factors, and differences, including discipline specific supervision for allied health professionals and local supervision for doctors.

**Conclusions:**

Strategies to enhance the experience of both allied health professionals and doctors in rural and remote areas include enabling career paths through structured training programs, hands on learning opportunities, quality supervision and community immersion.

**Systematic review registration number:**

PROSPERO CRD42021223187.

**Supplementary Information:**

The online version contains supplementary material available at 10.1186/s12913-022-08261-2.

## Background

People living in rural and remote areas are more likely to experience disease, injury and earlier death than those in metropolitan areas [1] and face more adversity than their metropolitan counterparts in accessing health services which has a negative impact on their health and wellbeing [2]. Workforce challenges including recruitment and retention of doctors, nurses and allied health professionals have negative impacts on the services that can be provided to rural and remote communities [3]. These challenges are complex and challenging to resolve [4, 5]. A range of initiatives have been introduced in Australia in an attempt to improve access to rural and remote health services through supporting the health professional workforce however these have predominantly focused on medicine [6]. Understanding the differences and similarities between the roles and the support strategies for each profession, and the success (or not) of the strategy in improving workforce recruitment and retention, may assist in addressing rural and remote health disparities.

There are a range of similarities and differences in the role and experience of doctors and allied health professionals in terms of clinical expertise, decision making, responsibilities and scope of practice, training and support mechanisms [7–10]. Rural doctors in Australia often have a rural upbringing, a positive rural experience at university or are attracted to the job opportunities and incentives offered by rural employers [11, 12]. Early career doctors undertake extensive post graduate training in order to specialise and advance their skills. Post graduate training can be done in rural areas and evidence suggests that this can have a positive impact on retention [5, 13, 14].

Allied health professionals also go to rural and remote areas for job opportunities, to be closer to family or partners, to gain diverse experience, or because of a desire to work in a rural area [15, 16]. Allied health professionals are not required to undertake post graduate training in order to practice autonomously, with varying requirements in terms of ongoing professional development, supervision and further training which does not necessarily relate to specialization or career progression [7, 17]. Retention of allied health professionals is influenced by the location of social supports, the availability of workplace support, high workloads, limited career advancement opportunities and opportunities available in metropolitan areas [15, 16, 18].

The demand for allied health services in rural and remote areas is growing with expanded funding mechanisms for people living with disability and chronic health conditions [19, 20]. Despite this increasing demand, workforce challenges continue to prevent rural people from accessing appropriate services locally to meet their needs [21].

Recent rural and remote health professional workforce systematic reviews have explored workforce challenges and have identified limited evidence for effective retention strategies [4, 11, 12, 16]. Buykx and colleagues [4] examined the impact of retention incentives for health professionals and found that although a range of factors were influential, these were multifaceted and complex. Interestingly the majority of the papers included in the review were based on the experience of doctors. Wakerman et al. confirmed the complexity of retention factors and made recommendations including; the need for quality education and training opportunities, safe and supportive work environments and consideration of clinicians’ personal needs [22], again this review mostly considered medical studies. Holloway, Donohue and Moore reviewed rural and remote recruitment and retention factors for doctors and identified the most significant factors were; rural background and experiences, access to rural training, professional support, support for partner and family and opportunities to integrate into the community [11]. Ogden and colleagues (2020) also found rural background and rural education experiences pre and post university were important predictors of recruitment and retention of rural general practitioners [12]. Couch et al. (2021) explored recruitment and retention influences for allied health and identified career opportunities, diversity of clinical work and workload, workplace supports and structures, rural background and experiences, location of partner or family and lifestyle factors as being significant.

With more rural and remote health workforce research focusing on doctors than allied health professionals, to date systematic reviews and commissioned reports mostly consider medical papers in their synthesised findings and recommendations [4, 5, 11–13]. There is a need to systematically explore whether the experience of early career allied health professionals and doctors is similar or different, upon which the development of evidence-informed workforce retention strategies can be developed.

While several reviews have explored the experience of health professionals working in rural and remote areas [4, 11, 12, 16, 22], no current systematic reviews compare the experiences of early career allied health professionals and doctors to investigate whether the experience are similar or different. Better understanding these similarities and differences will enable the development of recommendations for future workforce reforms.

This systematic review was undertaken to evaluate, synthesise and compare the experiences of early career allied health professions and doctors working in rural areas and the professional and personal factors that influence these experiences to identify similarities and differences of the two professional groups.

### Review questions

1. What are the experiences of early career allied health professionals navigating professional and personal factors when working in rural and/or remote environments?
2. What are the experiences of early career doctors navigating professional and personal factors when working in rural and/or remote environments?

## Methods

The systematic review was performed in accordance with the JBI methodology for systematic reviews of qualitative evidence [23]. The protocol was published [24] and the review was registered with PROSPERO (CRD42021223187). Theo Preferred Reporting Items for Systematic Reviews and Meta-Analyses (PRISMA) [25] guidance was adhered to throughout this review.

### Search strategy and selection criteria

A systematic literature search was conducted using Medline, CINAHL, Embase, Web of Science, and Informit. Grey literature was also searched using ProQuest Dissertations and Theses, Google Scholar and WorldWideScience.org. The searches were conducted between the 14th January and 2nd February 2021. Key search terms related to early career, the medical and allied health professions, rural and remote environments and experiences (qualitative research). Table 1 outlines the Medline search terms, these were adapted for the additional databases individual styles and phrasing requirements.

**Table 1:** Medline search

SSearch	Query	Records retrieved
1.	(“early career” or residency or “junior doctor*” or graduate* or registrar* or intern* or trainee*).tw,kf. OR “Internship and Residency”/	1082,351
2.	(physician* OR doctor* OR practitioner* OR GP*).tw,kf. OR (medical adj (personnel OR staff OR professional* OR worker*)).tw,kf. OR “allied health”/ OR rural generalist*.tw.kf. OR art therapist*.tw,kf. OR audiologist*.tw,kf. OR chiropractor*.tw,kf. OR (dietician* OR dietitian*).tw,kf. OR genetic counsellor*.tw,kf. OR music therapist*.tw,kf. OR nutritionist*.tw,kf. OR occupational therapist*.tw,kf. OR optometrist*.tw,kf. OR (orthotist* or prosthetist*).tw,kf. OR orthoptist*.tw,kf. OR pharmacist*.tw,kf. OR (physiotherapist* OR physical therapist*).tw,kf. OR podiatrist*.tw,kf. OR psychologist*.tw,kf. OR (radiographer* OR sonographer* OR radiation therapist*).tw,kf. OR rehabilitation counsellor*.tw,kf. OR (speech pathologist* OR language pathologist* OR speech therapist* OR language therapist*).tw,kf. OR ((health OR healthcare OR health care) adj (personnel OR worker* OR staff OR professional* OR workforce OR provider*)).tw,kf.	963,351
3.	((rural OR remote OR non-metropolitan OR nonmetropolitan OR regional) adj (communit* OR area* OR region* OR province*)).tw,kf. OR ((rural OR remote OR nonmetropolitan OR non-metropolitan OR regional) adj (health service* OR health care OR healthcare OR medical service* OR medical care OR workforce)).tw,kf. OR (rural OR remote OR non-metropolitan OR nonmetropolitan OR regional adj (setting* OR clinic* OR hospital* OR health service*)).tw,kf. OR rural Health/ OR rural hospital*, rural/ OR rural population/ OR rural health service*	142,677
4.	1 AND 2 AND 3	3211
5.	(((“semi-structured” OR semistructured OR unstructured OR informal OR “in-depth” OR indepth OR “face-to-face” OR structured OR guide) adj3 (interview* OR discussion* OR questionnaire*)) OR (focus group* OR qualitative OR ethnograph* OR fieldwork OR field work OR key informant)).tw,kf. OR interviews as topic/ OR focus groups/ OR narration/ OR qualitative research/	416,304
6.	7. 4 AND 5	575

Articles were included if: 1) they reported primary research, 2) used qualitative methodologies, 2) included early career doctors or allied health professionals, 3) focused on rural, regional or remote environments, 4) investigated experiences of the early career clinicians. Articles were excluded if they were not written in English, not based in high income countries, if they were published before 2000 and if they reported on the perspectives of students, managers, senior staff or supervisors rather than the early career professionals themselves. High income countries were defined using the World Bank criteria [26]. As there is no internationally accepted definition of rural and remote areas, papers where the author designated their study as focused on rural and remote areas were included. There is also no universal definition of allied health so the comprehensive list of included professions by Allied Health Professions Australia [27] was used to classify allied health professions included in the searches. Finally, there is no agreed definition of ‘early career’ in health professional literature. For the purposes of this review, doctors from their first year in the workforce up to specialty training programs were classified as early career while allied health professionals with 5 years’ experience or less or who were described as being early in their career were also included. The results of all searches were uploaded onto Covidence software.

### Data screening and extraction

Duplicates were removed and the titles and abstracts were screened by AD and SG, potentially relevant studies were retrieved in full and assessed against the inclusion criteria by all authors. Qualitative data was extracted by all authors using the standardised JBI data extraction tool [23] including details of the study methodology, methods, population, phenomenon of interest, country, setting, context, culture and outcomes relevant to the review questions. All decisions and discrepancies were made through discussion by at least two of the authors.

### Quality appraisal

Included studies were critically appraised for methodological quality using the standard JBI critical appraisal checklist for qualitative research [23]. All reviewers contributed to the appraisals and discrepancies were resolved through discussion with two reviewers. The JBI appraisal checklist identified whether the studies meet the criteria for high quality qualitative research across 10 questions. Reviewers judged the research based on what is presented in the paper and may not necessarily be a true indication of the study design. Given the anticipated small body of available literature, methodological quality was not used to exclude studies as the review team were keen to include all potential findings that could explore the review questions (Table 2 characteristics of studies).

**Table 2:** Allied health professions include in the review

art therapist audiologist chiropractor dental therapist dietitian exercise physiologist genetic counsellor	music therapist occupational therapist optometrist oral health therapist orthoptist orthotist prosthetist	perfusionist pharmacist physiotherapist osteopath podiatrist psychologist rehabilitation counsellor	radiation therapist radiographer sonographer social worker speech pathologist

### Meta-synthesis

Findings and their supportive illustrations were extracted from the primary studies. Findings were descriptions of the results reported by the authors that were relevant to the first two review questions. Illustrations were direct quotes from early career rural or remote allied health professionals or doctors. Where direct quotes from early career professionals were not provided by the author, the verbatim description of the finding were quoted as the illustrations. In cases where it was unclear whether the findings were reported by early career clinicians, they were not included in the review. The findings were extracted by the primary reviewer and confirmed by the secondary reviewers after thoroughly reading the papers.

Allied health and medicine findings were aggregated separately using the JBI meta-aggregative approach [23], which involved categorising the findings on the basis of meaning and quality against the research questions to generate a set of synthesised findings. Extracted findings were rated as either unequivocal (beyond reasonable doubt, supported with a direct quote), credible (result supported by an illustration from the author) or not supported (not supported with illustration of data). After analysing the allied health and medicine synthesised findings separately, they were then compared and contrasted to generate the discussion and recommendations.

The final synthesised findings were graded according to the ConQual approach for establishing confidence in the output of qualitative research synthesis [28]. Each synthesised finding from the review was presented along with the type of research informing it, a score of dependability and credibility and the overall ConQual score [28]. The synthesised findings relating to questions 1 and 2 exploring the experiences of allied health professionals and doctors are presented in the results of this review.

### Reflexivity

Reflexivity in qualitative research recognises the role that reviewers’ personal background, culture and experiences impact on how they shape, interpret and analyse research they undertake [29]. This review was conducted by a team of researchers with varied experiences and backgrounds and they engaged in robust discussion throughout the review process in relation to their own biases, experiences and perspectives and how these related to the research findings. AD is an occupational therapist, lecturer and PhD candidate investigating rural and remote allied health workforce and training initiatives, she has an extensive personal and professional background in rural areas. SG is also an occupational therapist and professor specialising in allied health service provision, driving, neurology and rehabilitation. NC is a speech pathologist and associate professor specialising in rural and remote and clinical education and CB is also a speech pathologist and professor specialising in allied health workforce and clinical education.

## Results

### Study inclusion and characteristics of included studies

**Fig. 1:**
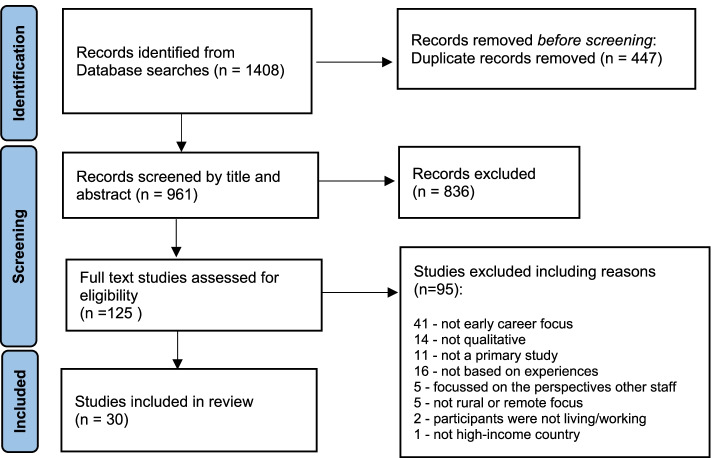
PRISMA Search results, study selection and inclusion process [25]

As detailed in the PRISMA flow diagram [25], the systematic literature search retrieved 1408 studies, with 30 meeting the eligibility criteria (see prisma Fig. 1). A total of 18 studies were excluded because it was unable to be determined how much experience the participants had or because the data was reported with participants with a wide range of experience levels and the early career findings were not able to be extracted separately.

The included studies were published between 2001 and 2020 and 21 utilised qualitative methodology, while 9 took a mixed methods approach with qualitative data that was able to be extracted. Fifteen studies reported using thematic analysis to analyse their data, five used descriptive analysis, four grounded theory, four constructivist interpretivist and there was one case study design and one longitudinal study. Of the 30 studies 25 used semi-structured interviews to collect data and the remaining utilised surveys, questionnaires and focus groups. All papers were peer reviewed, and no unpublished papers were included.

The majority of the studies were Australian [24], two were from Scotland and one each from Wales, Canada and New Zealand. Three studies reported including participants from remote areas and all papers included rural contexts. Of the 30 studies, 21 were based on the experience of early career doctors and 9 focused on allied health professionals. Of the allied health studies the following professions were included: dietetics/nutrition, exercise physiology, medical laboratory science, diagnostic medical imaging, occupational therapy, pharmacy, physiotherapy, podiatry, psychology, social work and speech pathology. Detailed information of the characteristics of the included studies is presented in Table 3.

**Table 3:** Characteristics of included studies

Author	Medicine/ Allied Health	Country	Population	Study design	Methods	Phenomena of interest
Bayley SA, Magin PJ, Sweatman JM, Regan CM [30]	Medicine	Australia	15 GP registrars enrolled in training	Qualitative Modified grounded theory	Semi-structured interviews, thematic analysis	Perceptions of compulsory rural GP vocational training program
Bonney A, Mullan J, Hammond A, Burns P, Yeo G, Thomson B, et al. [31]	Medicine	Australia	7 junior medical officers	Mixed methods Case study methodology	Semi structured interviews, pragmatic template analysis	Experiences of junior medical officers in metropolitan and rural emergency departments
Brown L, Smith T, Wakely L, Little A, Wolfgang R, Burrows J [32]	Allied Health	Australia	129 Allied health professionals undertook an undergraduate rural placement	Mixed methods, longitudinal study	Longitudinal survey, content analysis	Impact of rural immersive placement on longer term career outcomes
Campbell AM, Brown J, Simon DR, Young S, Kinsman L. [33]	Medicine	Australia	22 registrars and GPs upskilling in obstetrics in the last 5 years	Qualitative	Semi-structured interviews, thematic analysis	Factors influencing rural general practitioners and GP registrars to practise obstetrics
Cleland J, Johnston PW, Walker L, Needham G [34]	Medicine	Scotland	20 Trainee doctors	Qualitative	Focus groups and interviews, framework approach	Experiences and perceptions of trainee doctors working in remote and rural areas
Cosgrave C. 2020 [35]	Allied Health	Australia	74 managers, early career and experienced allied health	Qualitative constructivist-interpretivist	Semi structured interviews, thematic analysis	Influence of perceived work and personal factors on retention
Cuesta-Briand B, Coleman M, Ledingham R, Moore S, Wright H, Oldham D, et al. [36]	Medicine	Australia	21 junior doctors in postgraduate training	Qualitative descriptive	Semi-structured interviews, thematic analysis	Factors influencing the decision to pursue rural work among junior doctors
Cuesta-Briand B, Coleman M, Ledingham R, Moore S, Wright H, Oldham D, et al. [37]	Medicine	Australia	21 junior doctors in postgraduate training	Qualitative descriptive	Semi-structured interviews, thematic analysis	Junior doctors internal decision-making processes in relation to their career path understanding of how junior doctors
Devine S [38]	Allied Health	Australia	12 Occupational therapists	Qualitative phenomenological approach	Semi-structured interviews, thematic content analysis	Perceptions of rural occupational therapists regarding essential skills for rural practice graduates
Devine SG, Williams G, Nielsen I [39]	Allied health	Australia	17 past or present Allied Health Rural scholarship holders	Mixed methods	In-depth interviews, thematic analysis	Graduate recruitment outcomes and retention within a scholarship program.
Doyle C, Isles C, Wilson P [40]	Medicine	Scotland	14 rRural consultants and 23 junior doctors	Qualitative	Questionnaire and structured interviews, thematic analysis	Structure of teams, experience of role, perspectives of potential training pathway
Edwards SL, Sergio Da Silva AL, Rapport FL, McKimm J, Williams R [41]	Medicine	Wales	42 Junior doctors from the same medical program	Mixed methods, sequential exploratory	Online questionnaire and in depth interviews, thematic analysis	What influences students’ choices about either staying in, or leaving Wales, post-graduation?
Elliott T, Bromley T, Chur-Hansen A, Laurence C [42]	Medicine	Australia	30 Rural GP registrars	Qualitative	Semi structured interviews, thematic analysis	Comparison of pre and post rural rotation expectations and experiences
Gill SD, Stella J, Blazeska M, Bartley B [43]	Medicine	Australia	4 remote emergency medical trainees	Multi methods – observational study	Supervision documentation, pre and post semi scripted Interviews, thematic analysis	Experience of receiving remote supervision
Iedema R, Brownhill S, Haines M, Lancashire B, Shaw T, Street J [44]	Medicine	Australia	5 junior medical officers, 5 registrars, 2 consultants in one hospital.	Mixed methods.	Diary entries, content analysis	What are the barriers and facilitators of effective clinical supervision? Suggestions for improvement
Isaacs AN, Raymond A, Jacob A, Hawkings P [45]	Medicine	Australia	12 rural interns	Qualitative description framework	Semi structured interviews, thematic analysis	Exploring the job satisfaction, autonomy, training, social supports and mental health and wellbeing.
Keane S, Lincoln M, Smith T [46]	Allied health	Australia	30 rural allied health professionals	Qualitative study, grounded theory	Focus groups, thematic analysis	factors affecting recruitment and retention of rural allied health
Lee S, Mackenzie L. [47]	Allied health	Australia	5 new graduate rural occupational therapists	Qualitative	Semi structured interviews, thematic analysis	Attitudes and experiences of graduates working in rural areas
Malau-Aduli BS, Smith AM, Young L, Sen Gupta T, Hays R [48]	Medicine	Australia	20 International graduate registrars and 5 supervisors	Qualitative grounded theory	Semi structured Interviews over 2 phases, researcher notes	What impacts on registrars decisions to go to, to stay or to leave a regional, rural or remote area?
Martin R, Mandrusiak A, Lu A, Forbes R [49]	Allied health	Australia	12 Physiotherapists with 2 years or less experience	Qualitative general inductive approach	Semi structured interviews, thematic analysis	Perceptions of rural and remote practice and the influence of university training on preparedness for rural and remote practice
McKillop A, Webster C, Bennett W, O’Connor B, Bagg W [50]	Medicine	New Zealand	15 graduates who had studied for 12 months in regional and rural area	Mixed methods, descriptive design	Focus groups and interviews, thematic analysis	Attraction to rural area factors, career intentions and factors influencing these choices
Mugford BV, Braund W, Worley P, Martin A [51]	Medicine	Australia	2 interns who had undertaken a rural rotation, 2 supervisors, 1 hospital executive	Qualitative evaluation	Semi structured interviews, thematic analysis	The experience of rural interns undertaking a rural rotation
Myhre DL, Hohman S [52]	Medicine	Canada	29 resident doctors who had worked in a rural area for 4–8 weeks	Mixed methods	Survey, thematic analysis	The impact of rural rotations for post graduate medical training positions
Pandit T, Sabesan S, Ray RA [53]	Medicine	Australia	11 Junior and 9 senior rural doctors	Qualitative grounded theory	Semi structured interviews, thematic analysis	Perceptions of training needs of rural doctors
Peel R, Young L, Reeve C, Kanakis K, Malau-Aduli B, Sen Gupta T, et al. [54]	Medicine	Australia	79 GP registrars, managers, supervisors, consumers and practice staff	Qualitative2 phases	Semi structured interviews and focus group, thematic analysis	Attractors and barriers for GP registrars to train and GP supervisors to work in rural and remote communities
Smith DM [55]	Medicine	Australia	19 rural Junior and senior doctors, educators, directors, medical administrators	Qualitative exploratory	Semi structured interviews, thematic analysis	Issues and difficulties faced by junior doctors with bonded scholarships
Steenbergen K, Mackenzie L. [56]	Allied health	Australia	9 new graduate rural occupational therapists	Qualitative	Semi structured interviews, thematic analysis	The experience of professional support for occupational therapists
Thackrah RD, Thompson SC [57]	Allied health	Australia	3 Occupational therapists and speech pathologists, one health science graduate	Qualitative	Semi structured interviews, thematic analysis	Long term impacts of rural placements, the experience of working rurally
Walters L, Laurence CO, Dollard J, Elliott T, Eley DS [58]	Medicine	Australia	18 rural GP registrars	Qualitative grounded theory	Semi structured interviews	Exploring the resilience of rural GP registrars and strategies used to maintain resilience
Wearne SM [59]	Medicine	Australia	5 Registrars who had completed a 6 month remote rotation	Qualitative	Structured interviews, content analysis by question	Factors in the interaction between GP registrars and supervisors impact on the quality of registrar learning

### Methodological quality

The methodological quality of included studies using the JBI critical appraisal checklist for qualitative research [23] is summarised in Table 4 and full details of the appraisals are outlined in the appendices. One study had limited methodological detail described and was deemed to be of low quality (4/10), however the other studies were rated moderate or high quality, five studies scored 7/10, 13 studies scored 8/10, seven studies scored 9/10 and four studies scored 10/10. Most studies did not locate the researcher culturally or theoretically and eighteen of the 30 studies did not state the influence of the researchers on the results. All studies were included for analysis.

**Table 4:** Quality of selected studies, number of studies meeting JBI critical appraisal checklist criteria

	Yes	No	Unclear
1. Is there congruity between the stated philosophical perspective and the research methodology?	25	1	4
2. Is there congruity between the research methodology and the research question or objectives?	29		1
3. Is there congruity between the research methodology and the methods used to collect data?	30		
4. Is there congruity between the research methodology and the representation and analysis of data?	29		1
5. Is there congruity between the research methodology and the interpretation of results?	29		1
6. Is there a statement locating the researcher culturally or theoretically?	4	26	
7. Is the influence of the researcher on the research, and vice- versa, addressed?	12	18	
8. Are participants, and their voices, adequately represented?	30		
9. Is the research ethical according to current criteria or, for recent studies, and is there evidence of ethical approval by an appropriate body?	28	2	
10. Do the conclusions drawn in the research report flow from the analysis, or interpretation, of the data?	30		

### Meta-synthesis

An analysis of the 30 papers resulted in 331 findings (202 medicine and 129 allied health) across 23 categories (13 allied health and 10 medicine). The categories were integrated into three synthesised findings which were common to both professional groups (see Table 5 for synthesised findings and categories). In terms of credibility, 231 findings were unequivocal, 97 were credible and none were not credible (see appendices for full details). Each of meta syntheses ConQual scores were low overall as they contained a mixture of credible and unequivocal findings as well as high and moderately rated appraisals.

**Table 5:** Synthesised findings and categories

Synthesised findings	Allied health categories	Medicine categories
Making a difference through professional and organisational factors	1.1.1 Supervision 1.1.2 Manager support 1.1.3 Human resources 1.1.4 Workplace culture	2.1.1 Supervision 2.1.2 Human resources 2.1.3 Workplace culture
Working in rural areas can offer unique and rewarding opportunities for allied health professionals	1.2.1 Broad clinical opportunities 1.2.2 Career opportunities and challenges 1.2.3 Opportunities for Autonomy 1.2.4 Learning opportunities 1.2.5 Professional development opportunities	2.2.1 Broad clinical opportunities 2.2.2 Career and specialisation opportunities and challenges 2.2.3 Autonomy and professional identity 2.2.4 Hands on learning opportunities 2.2.5 Training opportunities
Personal and community influences make a difference	1.3.1 Family and partner influences 1.3.2 Community influences 1.3.3 Accommodation influences 1.3.4 Professional personal boundaries	2.3.1 Family and partner influences 2.3.2 Community influences

In this section we present the results by describing the synthesised findings and associated categories outlined in Table 5 and at the end of each category the relevant quotes are presented in tables.

### Making a difference through professional and organisational factors

Early career allied health professionals and doctors working in rural areas reported varied experiences based on a range of professional and organisational factors including supervision, human resources and workplace culture. Allied health professionals discussed manager supports but this was not a finding for doctors.

#### 1.1.1 and 2.1.1 Supervision

Having access to adequate clinical support determined how supported allied health professionals felt in developing their skills and expertise. Clinicians experiencing limited supervision reported challenges in developing confidence and diverse skills. Without supervision, allied health professionals reported not knowing who to ask questions of or seek support from and feeling isolated in making clinical decisions. Allied health professionals who were receiving regular, supportive supervision described developing confidence and skills to work through challenging situations.

Many rural areas offered doctors supportive workplaces with good access to clinical supervision and informal supports. When a senior doctor was available for advice or guidance, junior doctors were likely to feel confident managing their caseload. Doctors valued access to feedback about their performance and formal and informal support opportunities. Early career doctors generally found the senior doctors to be good role models and rural services enabled them to be directly supported by consultants, rather than other doctors in training. Having high levels of support resulted in early career doctors feeling confident to ‘have a go’ knowing the senior doctor would be available if needed.

Early career doctors receiving remote or less frequent supervision in remote health services reported experiences of isolation and stress. For some doctors it was challenging to access adequate supervision in rural areas, reporting limited opportunity for informal support, with early career doctors contacting senior doctors to solve specific clinical problems rather than for broad skill development. With limited supervision and support, doctors reported feeling stressed, isolated, overwhelmed and lacking in confidence in their own skills.

**Table:** 

Allied health	Medicine
1.1.1 Positive supervision and support 2.1.1 Positive supervision and support	
*“More support meant more freedom to ask questions and increased confidence. Opportunities to discuss practice dilemmas as part of professional support decreases anxiety.” pg 163* [56] *“having the opportunity to bounce things off my colleagues and discuss difficult circumstances with my seniors [helped me through difficult days]....the senior support and collegial support has been amazing”. pg 4* [57]	*“you have to deal with everything that walks in the door. But you are paired with a consultant on the day. You basically run your assessment with them and see if they are happy with your plan, and for any instrumental deliveries or complicated issues you contact them to come in.” pg 668–669* [33] *“I was really lucky ‘cause I went to such a supportive practice. I think, potentially, if it’d not been as supportive and I hadn’t had that backup so frequently available, it could have been more stressful.” Pg 83* [30]
1.1.1 Challenging or absent supervision and support 2.1.1 Challenging or absent supervision and support	
*“None of the study participants (including those working with other occupational therapists) reported being involved in structured supervision with another occupational therapist... Less support caused difficulty developing confidence, especially in a newly created position” pg 162–3* [56] *“It’s different talking to someone on the phone than having them there, in the office when you want them. Like you could ring the adviser and she won’t be able to get back to you if you ring her one morning, till the following afternoon. Often you’ve needed to make a decision by then. So you’ve had to make one anyway.* “*pg 40* [47]	*“Yeah, there’s been times that I’ve been very stressed and upset, but not sure who to go to. I think that’s one thing that internship really lacks is someone who is there to look out for us interns.” pg 249* [45] *‘Unable to contact any senior staff regarding sick patient abandoned, overwhelmed’ ‘Registrar did not listen & was very dismissive poor advice given, felt very unsupported’ ‘Mocked by another registrar about previous mistake on patient insulted, unhelpful’ [Field notes] pg 290* [44]

#### 1.1.2 Manager support

Support from a manager influenced allied health professionals experiences in rural areas and this was reported as a different role to the clinical supervisor. Manager support findings were not found in the medical papers. It is assumed that doctors received both supervision and management support from a discipline specific leadership role while allied health professionals were often supported by a manager working across multiple disciplines.

Some allied health professionals reported managers helped them transition into the work role and build confidence. When managers were not supportive, allied health professionals felt less satisfied in their workplace and were less likely to intend to stay in rural areas.

**Table:** 

Allied health	
1.1.2 Positive support provided by manager	
*“Anything I need, anything I have to run by them, they make the time for me and X [name of manager] really gives me a lot of confidence in my abilities. She’s like, ‘Why are you worrying about this? It’s exactly what I would have done.’ ‘Of course, you’re on the right track.’ ‘If you forgot to ask a question [to a patient], you can go back and see them, tomorrow, can’t you?’ or ‘It’s just no fuss.’ I’m stressing about these things that I was made to stress about on placement which I don’t ever stress about here, it’s completely different.” pg 13* [35] *“My manager creates the environment and I feel like... she’s the very key reason the staff that I work with are here and a very key reason for why I love to work here.” pg 11* [35]	
1.1.2 Challenging or absent support from manager	
*“My boss is extremely unorganised, trying to organise time off is a nightmare unless you are [in a] senior [role]. I also feel it is not on a first apply, first granted basis. I also feel my boss is unapproachable.”* [32] *“The perceived absence of a supportive manager was sharply felt and described as having negative impacts on job satisfaction: ‘[Early career is] not really easy. I personally don’t advise new grads to work in rural anymore. I think they need support and no matter how much promise they get, I got a lot of promises but I didn’t get a lot of support.” pg 13* [35]	

#### 1.1.3 and 2.1.2 Human resources

Human resource factors were reported by both allied health and medical professionals. Findings in allied health papers focussed on the challenge of working with short term contracts, limited notice of contract extensions and lengthy recruitment processes. Human resource processes impacted on allied health professionals’ satisfaction at work. Short contracts were reported as a retention barrier with clinicians having limited job security.

Human resource findings for doctors included inconsistent expectations, challenges with contracts and job opportunities, quality of provided accommodation and inadequate pay. Human resource process issues impacted negatively on early career doctors experience in rural areas.

**Table:** 

Allied health	Medicine
1.1.3 Human resources	2.1.2 Human resources
*“So the HR process took a long time to come through....Maybe I interviewed in early Feb then, because I remember starting on the [late date in] March.... as that was as soon as HR could onboard me... So I remember like it made me doubt myself..... and I thought how could I have not gotten this job?” pg 12* [35] *“I hope to still be working at the hospital in two years’ time but I do want permanency.... I’d love to stay (where I am) but I’ll leave, only because of the permanency issue; this is a contract position.”pg 4* [57]	*“Yeah, I think there’re difficulties between DHB expectations, college training expectations, university expectations, RMO [resident medical officer] expectations...” pg 11* [50] *“Accommodation could be better. There’s no Internet access at all, so we struggle to do our DOTS modules [compulsory online learning]. We don’t even have a telly (television) that works....these home comforts are actually fairly important.” pg 480* [34]

#### 1.1.4 and 2.1.3 Workplace culture

Workplaces who embraced early career allied health professionals, finding ways to make them feel welcomed, included and appreciated, were enablers for clinicians overall feeling satisfied. Allied health professionals reported enjoying working with their colleagues, who were approachable, non-judgemental and supportive.

The workplace culture in rural areas for doctors was generally reported to be positive, and doctors felt like they were part of a supportive team. Taking the opportunity to make a difference to a rural community and feeling accountable to their community was a positive experience for early career doctors. For some doctors the workplace culture had a negative impact on their experience in rural areas, reports of having too much responsibility early in their career had a negative impact on confidence, stress and intention to stay in a rural area.

**Table:** 

Allied health	Medicine
1.1.4 workforce culture	2.1.3 workforce culture positives
*“Rural and remote colleagues were seen to be ‘friendly’, ‘laid back’, ‘sociable’ and ‘supportive’.” pg 448* [49] *“I felt really welcomed. As soon as I got here, they made sure I was okay, got to know me, had a welcome dinner. Y [staff member’s name] organises all of the social events for X and that was a good opportunity to get to know them outside of work, you talk about different things.” pg 17* [35] *“I enjoy working with my colleagues.” “I enjoy it and I like the location and the people.” “I like the way the hospital works, together with all allied health professionals and all hospital staff.”* [32]	*“It’s different in that it’s usually only you and one other doctor and two or three nursing staff so you really feel very involved in the process and you actually really feel like you’re making a difference.”pg 479* [31] *“They lacked resources... but they have a really good work culture which I thought was really, really amazing.” pg 247* [45]
	2.1.3 workforce culture challenges
	*“There was too much. It was quite stressful. The demands of rural practice are probably too high the stress and.... responsibility [and the] considerable personal cost associated with that. I guess a lot of people do it, survive and cope but I can’t see myself doing it at that sort of level.” pg 85* [30] *“I’ve had to deal with all sorts of horrendous situations... I’m glad I’ve done it in a way but I think it would have been nice to have got that experience without being sent to the middle of nowhere by myself” pg 3* [55]

### Working in rural areas can offer unique and rewarding opportunities for allied health professionals and doctors

Allied health professionals working in rural areas are afforded a range of opportunities early in their career that they may not experience in other settings including high levels of autonomy and problem solving. Access to professional development activities are highly valued as they develop their professional identity, skills and confidence.

Early career doctors also experience a broad range of clinical opportunities in rural areas, the work is complex, and the level of autonomy is high compared to work in a metropolitan area. Training and skill development is imperative in these environments but at times is difficult to access. Specialisation opportunities are unique in rural areas with general practice being the most common option discussed.

#### 1.2.1 and 2.2.1 Broad clinical opportunities

In rural areas allied health professionals are afforded a broad range of clinical experiences in a range of complex settings. A range of clinicians reported these experiences as being positive, satisfying and enabling the development of confidence and skills early in their career, that may not have been possible outside of a rural environment.

Early career doctors have the opportunity to work with a wide variety of clinical cases with high levels of complexity in rural areas. Rural doctors develop broad ranging skills, are less reliant on specialists for assistance, can manage complex situations and have the opportunity to pay more attention to rural people to meet their needs than their metropolitan peers.

**Table:** 

Allied health	Medicine
1.2.1 Broad clinical opportunities	2.2.1 Broad clinical opportunities
*“The diversity of duties that needed to be performed was seen as challenging ....the assorted needs of the client groups ....The importance of having administrative skills and broader management skills was also discussed. Although identified as challenges, these issues were also seen to add to the attractiveness of rural practice.” pg 207* [38] *“I think the biggest thing is the diversity of the case load. On placement it was a set discipline or a set ward that you’d be on and even as a new-grad working in those areas....Whereas out here, I can go from an Ortho, to a MSK, to a Paeds, you know....even in one day it’s a very different case load.” pg 448* [49]	*“[The rural hospital] was the complete range of patients so I saw lots of patients who didn’t need any treatment at all, right through to patients who had a triage category of one and had either died or were dying at the time. But it’s very unusual for the intern to see a patient who is severely ill at [the metropolitan hospital] because the registrars usually see those patients.” pg 480* [31] *“The variety in just one day is incredible I think. I compared it to what my urban GP placement was like in sixth year and there’s no way that we would have been doing the variety of things. Yes, it’s kind of hard to explain but I was really just impressed with how many different things I could see just in one day.” pg 9* [42]

#### 1.2.2 and 2.2.2 Career and specialisation opportunities and challenges

Career development opportunities are important to allied health professionals, having positive opportunities for growth in rural areas was reported by some participants. When clinicians experienced challenges accessing career development or specialisation opportunities, this had a negative impact on their experience and intention to stay in a rural areas.

Early career doctors report having positive career opportunities in rural areas. Individuals who are interested in pursuing a general practice or rural generalist specialisation are afforded good opportunities in rural areas. Junior doctors in rural areas have access to timely support from consultant doctors and other team members with teams working closely together as a result of small, rural teams working collaboratively.

In some areas, early career doctors experience limited opportunities to specialise their skills unless they are interested in general practice, anaesthetics or another specialty training offered at the rural service. When doctors are interested in other specialist training they generally need to leave rural areas to pursue training positions in metropolitan areas despite a desire to work in a rural context.

**Table:** 

Allied health	Medicine
1.2.2 Career opportunities	2.2.2 Career and specialisation opportunities
*“OK, now I’m here, where’s the next step up?” And the career opportunities are all out here, they’re not back in the city.” pg 8* [46] *“I hope to regain employment when my contract finishes as the region has good capacity for growth.” (rural/remote based graduate, physiotherapy)* [32]	*“..it’s a small little hospital where you had open access for the undifferentiated patients that presents with a problem and it’s got a significant emergency, significant outpatients segment. It’s got obstetrics and a huge Indigenous population. It suited me.” pg 3* [58] *“So, I guess at this point I want to be a generalist. I like a bit of everything, it keeps it interesting, it keeps it fresh” pg 6* [37]
1.2.2 Career challenges	2.2.2 Career and specialisation challenges
*“Whilst I enjoy the rural lifestyle and experience. As a new graduate, I am limited with opportunities to further my career … .. I am moving somewhere where they have the resources to provide me with better support and opportunities.” pg 9* [32] *“Professionally and clinically my particular interests make it a bit difficult to work in regional areas....I loved working in [the country] and that’s why I stayed so long, but the thing that really drew me home last month [to the city] was that I wanted to gain more experience in a very specific area... you don’t get the opportunity to do that in rural areas”. pg 6* [57]	*“There aren’t training jobs in the rural hospitals, apart from GP training, which is not what I want to go into straightaway. So it’s actually quite frustrating, because I’ve loved these two years, but there’s nothing to go into afterwards, so that’s why I’m going away.” pg 479* [34] *“… unless you specifically want to be that rural GP, there’s firstly no pathway. And two, it’s not only not encouraged, it’s almost frowned upon. I find it amazing because the whole time I was in rural areas people talk about how much they’re trying to bring people rurally. When I look at it I kind of see a lot of closed doors.” pg 7* [36]

#### 1.2.3 and 2.2.3 Opportunities for autonomy and developing professional identify

Allied health professionals have the opportunity to work with high levels of autonomy and to be creative in their practice. High levels of autonomy and clinical complexity can however be very challenging for early career clinicians who reported experiences of high workloads, stress, limited support, long hours and burnout while working in rural areas. These factors were linked to clinicians choosing to leave rural practice.

Early career doctors are also afforded a high level of autonomy in rural areas which can be a positive or challenging experience. Having the opportunity to make clinical decisions in practice is daunting but also a chance to maximise skill development. Developing a professional identity in rural areas was reported to be complicated for early career doctors. They are required to reflect on their practice, build resilience and manage their self-care while undertaking training on the job and managing busy caseloads. It is imperative that they can recognise their limitations and know when and how to seek assistance. This was reported as challenging when early career doctors did not feel prepared and confident for rural practice.

**Table:** 

Allied health	Medicine
1.2.3 Opportunity autonomy	2.2.3 Opportunity for autonomy
*“I like being in a rural area because I have the independence and ability to structure things the way I want to. I love the autonomy, the travel and seeing all these different things.” pg 208* [38] *“Less support meant more responsibility to seek out answers to questions, develop skills, become more independent and facilitate creativity.” pg 163* [56]	*“Increased sense of autonomy in clinical decision making and in particular felt they had the opportunity to develop and implement patient management plans” pg 2* [51] *“You stop and question whether you actually need to speak with a consultant...and now I’m back here [teaching hospital] I ask less for advice …*. I*t forces you to step up to the next level”. pg 449* [43]
1.2.3 Negatives of autonomy	2.2.3 Medicine developing professional identify
*“I’m doing two jobs and have been doing for two and a half months. Recruitment is happening and it’s going, and I hit the wall and my manager said, “Keep on going,” and I said, “Can you just acknowledge how much extra – all you need to do is acknowledge it...” pg 5* [46] *“I don’t think I’ve necessarily made the wisest decision with what I’ve done (becoming a sole therapist). I’ve made a decision which I certainly benefited from, but professionally and personally it’s been a hard slog …*.” *‘pg 42* [47]	*“I think it is about fostering supported practice and this is a particular time of vulnerability in terms of support....the movement from hospital-based practice to being a new person in community-based practice.” pg 669* [33] *“I call my boss before each shift, my supervisor, and say, I’m on tonight because he gives me telephone back up which I rarely use, but I actually like to know that he knows that I might be calling him.” pg 10* [42] *‘You could finish your internship and it could be your first week in your JHO year* *and be sent to a rural site and you’re acting as a PHO or SMO, or something,* *which I think is terrifying and a bit inappropriate.’ Pg 4* [53]

#### 1.2.4 Learning opportunities and 2.2.4 Hands on learning opportunities

While transitioning to working in rural areas, allied health professionals experienced a steep learning curve while managing busy workloads, some allied health professionals experienced this steep learning curve as a positive opportunity for skill and confidence building. Experiences of being thrown in the deep end were also reported, as well as needing time to adjust to the diverse caseloads. Clinicians reported limited resources and services in rural areas was challenging, with a need to be creative in terms of how clients’ needs could be met.

The opportunity to be involved in hands on patient care and being thrown in the deep end early in their career enabled junior doctors to build skills and confidence that they may not have had in a metropolitan health setting. Rural doctors also have the opportunity to follow patients from the community to hospital which was reported as being a positive learning and practice experience. They also feel like they are making a difference to the community, they get to know their patients well and the patients are appreciative of services they receive. Early career doctors also reported high workloads with limited cover available, they were also required to travel long distances to provide services, work on call out of hours and in some situations they had limited opportunity to practice skills due to the types of presentations they were exposed to at work.

**Table:** 

Allied health	Medicine
1.2.4 Positive learning opportunities	2.2.4 Positive hands on experiences
*“You’ve really got to embrace it. Think of it, like an opportunity to learn and experience a lot of different...a variety of patients from various demographics and backgrounds.” pg 448* [49] *“Overall, participants felt they had gained many skills as a result of their rural practice that would not have been gained outside the rural setting.” pg 208* [38]	*“You get to do a lot more clinically, you don’t get this hands on experience in a less remote setting” 3 pg 9* [40] *“So you get to look after the patient in general practice and then if they’re sick, you look after them in hospital. That was great.” pg 9* [42] *“Great hands on clinical and operative skills rotation” pg 5* [52]
1.2.4 Challenging learning conditions	2.2.4 Challenging hands on experiences
*“Study participants often found that it was their responsibility to try to enhance the available resources. As new graduates they had not expected this responsibility and felt unprepared and overwhelmed.” pg 41* [47]	*“I’ve had to deal with all sorts of horrendous situations... I’m glad i’ve done it in a way but I think it would have been nice to have got that experience without being sent to the middle of nowhere by myself” pg 3* [55]

#### 1.2.4 Allied health professional development opportunities and 2.2.4 Medicine training opportunities

Rural allied health professionals are required to maintain their professional competence for registration or for membership to professional associations [17] but they are qualified to work autonomously on graduation. Findings from this review indicate rural allied health professionals have mixed experiences in accessing professional development activities. While this was highly valued and enabled the development of clinical skills and confidence, a range of barriers to professional development were also reported, including heavy caseloads, limited cover, funding and travel challenges.

Early career doctors undertake structured training programs as a requirement of their professional registration and competence development. In rural areas, doctors reported a wide range of experiences in terms of their access to training. For many, working in rural areas while training offered positive opportunities for learning and development. The programs were high quality, accessible, services were accommodating of training needs and there were hands on opportunities for integrating learning in practice. Choosing a general practice or rural generalist training pathway gave early career doctors broad and in-depth learning and skill development and the ability to solve problems. Challenges accessing training were also reported including difficulties with technology, travelling long distances to access training, employers not prioritising training, funding limitations, heavy workloads and inadequate cover. In these instances, the early career doctors felt their training needs were not being met.

**Table:** 

Allied health	Medicine
1.2.4 Positive professional development opportunities	2.2.3 Positive training opportunities
*“Yeah, good training opportunities, quick training opportunities, you’re able to get training quickly here as in compared to bigger metropolitan cities [where] it takes a while.” pg 15* [35] *“… work was supportive of me taking the time off for leave and paid for the course as well, which I really didn’t expect. Which was really nice ….” pg 14* [35]	*“..most doctors thought that the resources available at smaller hospitals were adequate to meet their training needs, and some even spoke of the benefit to their clinical reasoning of having limited access to diagnostic technology, where having to ‘make do’ with minimal equipment resulted in their becoming more independent thinkers.” pg 5* [36] *“The consultant did weekly teaching; actually twice weekly teaching; So, after work hours, he would do a non-formal tutorial with the registrar and the intern, and I thought that was good...”pg248* [45]
1.2.4 Challenges with professional development	2.2.3 Challenges with training
*“Large caseloads and the inability to find locums prevented attendance at professional development events. Travel distances and overall expense were also barriers.” pg 207* [38] *“Difficulty accessing useful continuous professional development... travel and time burden to attend educational sessions in metropolitan centres … need for better access to training opportunities available locally..” Pg 163* [56]	*“In some practices, tutorials were not given priority on the timetable and so did not occur, or else were held outside of work hours” pg 8* [59] *“it is far away from everywhere, so you have got to add a whole day for travel just because of the time of flights and the cost” pg 5* [54]

### Personal and community influences make a difference

Personal factors play an important role in allied health professionals and doctors experience of working in rural areas. Allied health findings related to the location of family and friends, integration into the community, access to housing and professional personal boundaries. Early career doctors described the needs of their partner and children as vitally important when living and working in a rural area. They generally found the community to be very welcoming and there were a range of lifestyle benefits to living in rural areas.

#### 1.3.1 and 2.3.1 Family and partner influences

Early career allied health professionals experienced personal challenges when their family or partner did not live close by. High staff turnover with colleagues regularly leaving was also reported as a personal challenge for maintaining social networks. Having a partner living locally was reported as a reason to stay in the rural area and conversely clinicians were planning to leave to be closer to family or a partner in the future.

Many early career doctors reported their family or partner's needs as significant factors to consider when working in rural areas. Family or a partner living in the same location enabled doctors to feel socially supported. Not having family living nearby was a significant challenge. If significant others were close enough to visit on weekends, this was seen to be favourable and had a positive impact on overall satisfaction. For doctors who had brought their family to the rural area, their partners sometimes faced challenges accessing work and social supports and there were also difficulties accessing childcare or education opportunities for children.

**Table:** 

Allied health	Medicine
1.3.1 Family and partner influences	2.3.1 Family and partner influences
*“My family is in the city, so that’s been the hardest thing, being so far away. It might be a factor in making a consider moving, but we’ll see how that goes” pg 5* [46] *“I will move closer to my partner at some stage as they seek different employment opportunities, but I am hoping to stay working regionally or rurally.” pg 9* [32] *“I decided to stay around instead of moving away because my boyfriend is here.” pg 6* [46] *“Personal factors such as marrying a person from the area and having friends or family in the area also had an impact” pg 207* [38]	*The most important people in my life is just my family, my wife and kids. They are like shock absorbers for you and sometimes you have ups and downs and stress, and sometimes something doesn’t go well you get upset and that is part of work and life. So you need some like you need to unwind your stress, so you need your partner just to sit and talk and de-stress yourself. pg 11–12* [48] *“In terms of professional concerns, and the separation from my wife, she was very, very supportive. We worked out that it was good, [my rural placement] was only an hour-and-a-bit from where we lived in Adelaide so weekends where I wasn’t on call in [my rural placement] I’d go down to Adelaide and vice versa … ..” pg 10* [42]

#### 1.3.2 and 2.3.2 Community influences

Integration into the community helps allied health professionals feel welcome, some participants reported they felt welcomed and involved in community activities when they arrived in a local area. Having the opportunity to be involved in sport and social networks enabled early career allied health professionals integrate into the community.

A range of early career allied health professionals experienced challenges integrating into the local community. They felt like outsiders in the rural area and found community activities were difficult to find or the activities did not suit their interests. Some participants reported feeling unwelcome at community activities while others reported difficulties making friends in the local area. These challenges had a negative impact on their overall experience.

Junior doctors experience of feeling welcomed in the rural community had a positive influence on their experience in rural areas. Doctors reported a positive of working rurally was being well known, seeing patients out and about and feeling part of the community. Lifestyle factors were also positive including social outlets, outdoor activities, short commute times and a community atmosphere. In contrast, some doctors found integrating into the community challenging and experienced social isolation in rural areas and everyone knowing each other.

**Table:** 

Allied health	Medicine
1.3.2 Community influences positive	2.3.2 Community influences positive
*“I really like the community support and spirit. Even outside of work the community is really good and it’s easy to meet people. The community focus rather than the medical model focus is great.” pg 208* [38] *“Within one or two weeks he was offering if I wanted to play on a social touch team....Everyone only had positive experiences about going out there (rural town), they all kind of tell you the things that you can do on the weekends!” pg 448* [49]	*“very nice lifestyle. It’s not as busy, not as fast, not as crowded, everything is just nice. You know, you’ve got short ways everywhere. You don’t have to drive so far. You get parking spots everywhere. You don’t have to pay for everything. The nature is easily accessible. The people are usually relaxed and nice. Hospitals are small, you know, more working in a family than like in the big [urban] Hospital.”pg 13* [48] *“I just made the most of it. I really enjoyed going from a big city to being in the outdoors, learned how to sail, went hiking lots, and just made the most of it.” pg 480* [34]
1.3.2 Community influences negatives	2.3.2 Community influences negatives
*“‘getting out into that wider community has been difficult’ pg 41* [47] *“I think in the country towns is if you’re not sort of in the football, netball, then it’s harder I suppose to make those connections outside of work and get to know the people.” pg 19* [35]	*“...it can be quite isolating as well.... if you’re not from there, you’ll tend to make friends who are related to the medical side of things, and there’s not so much going on in the city as perhaps in bigger cities, so it’s kind of hard to get away from it....But medically, I’d say it’s –it’s good in that, I thought it was ....quite captivating.” pg 6* [41] *“Adjusting to living in small communities... those accustomed to living in big cities found it quite peculiar in towns where everybody knew everybody, and everyone knew everyone else’s business” pg 5* [55]

#### 1.3.3 Accommodation and commuting influences

Allied health professionals reported challenges with accommodation especially finding somewhere suitable to live and not being financially supported with moving or living costs. Some participants were offered rooms with colleagues which helped them feel welcomed. Allied health professionals living in the city and commuting to work in a nearby rural area each day or at the end of the week, were less likely to getting involved with the local community as they were not investing time into integrating into local activities or networks.

**Table:** 

Allied health	
1.3.3 Accommodation	
*“Access to appropriate and affordable accommodation was important and assistance in finding accommodation was recommended as well as having access to financial support for accommodation and relocation costs.” pg 7* [39] *“I just couldn’t find anything. I just thought, ‘I can’t find anything that fits the bill’ … ..My working hours are anywhere between 7 and 5, so it’s just, it was impossible to even to get to a real estate office to say, ‘I’m looking for a property, I want some support’ ... I’d have friends going to inspections for me.” pg 17* [35]	
1.3.3 Clinicians commuting to work	
*“I would love to be closer and I have close bonds with people [here] but there is still the [distance] barrier that separates you from developing... things further. And a lot of other people are not from here, so they’re most likely to go back home [straight after work] anyway …”* [35] *“There was a couple of people there who just weren’t interested in any of the regional stuff, unless it was open after hours on a Monday to Thursday because ‘we’ll only be here for one year and we’ll be going to Melbourne every Friday night and coming back on Monday morning’.”* [35]	

#### 1.3.4 Professional personal boundaries

Working in rural areas present allied health with challenges of seeing clients in the community and having their personal boundaries challenged. They recognised there were benefits to seeing clients progress over time but also found the challenge of being known in the community and not being able to switch off after hours was challenging.

**Table:** 

Allied Health	
1.3.4 Professional and personal boundaries	
*“I’d have to sort of, deal with, like, people interacting outside of a professional environment? I saw another one of my patients at the pub. We were drinking, and I was like ‘this is kind of weird’ so I don’t know, I found that actually quite hard, like how much, how do I even, interact with them?”* [49] *“I’d walk around and people would recognise me as the new physio and essentially. I was filling up my car at the petrol station and a guy came over and said ‘Are you the new physio?“pg 447–448* [49]	

## Discussion

This review sought to better understand the experience of early career allied health professionals and doctors in rural and remote areas. We found 30 qualitative papers that met the inclusion criteria that explored a range of experiences. Studies were heterogenous in terms of sample sizes, locations and methodologies, although most were based in Australia. The meta-synthesis identified three key synthesised findings shared across the two professional groups relating to professional and organisational factors, professional opportunities and personal and community influences.

The synthesised findings are consistent with previous systematic and scoping reviews exploring workforce challenges for health workers in rural and remote areas [4, 11, 16, 60]. These reviews also identified opportunities and challenges around supervision and support, training, career advancement, diverse work opportunities, personal factors and community integration. This review explored the experiences of allied health professionals and doctors separately in order to identify the similarities and differences for both groups. Both allied health professionals and doctors valued the supervision and support they received with reports of increasing skills and confidence from high quality support. When supervision and support was felt to be inadequate, both groups reported challenges with confidence, being overwhelmed and lacking satisfaction in their roles. These findings build on previous research [60] outlining support challenges for health workers in rural areas. Remote supervision was reported to be challenging when there was limited opportunity for hands on, informal and timely support. The early career doctors reported an advantage of working in rural areas included being directly supervised by consultants and senior doctors rather than other doctors in training, which they may have experienced in metropolitan centres, this finding was not widely reported in previous reviews. A range of allied health professionals reported not receiving profession-specific supervision. In contrast, despite some doctors accessing support remotely, they all appeared to have a supervisor to call on. Allied health professionals reported being operationally supported by a line manager who was not necessarily from their discipline, in many instances the line manager’s support was an enabler for positive job satisfaction and professional development, but some clinicians reported negative impacts from non-supportive line managers. Doctors did not mention the role of a separate line manager to their supervisor and appeared to be reporting to senior doctors predominantly. This difference may relate to the varied disciplines of allied health and a lack of available discipline specific supervisors and also the different employment and renumeration structures for both groups.

Both allied health and medicine papers reported on the diverse caseload that rural practice afforded. They also commented on positive workplace cultures and small supportive teams who worked together effectively. Both groups reported the caseload in rural areas was often complex and the workload was heavy. Early career medicine findings included the opportunity for hands on learning, autonomous practice and the following of patients from the community to hospital in rural locations, which was not available in metropolitan areas. Early career doctors reported not feeling adequately prepared for rural practice and at times the level of autonomy afforded to them was inappropriate for their stage of learning. In comparison, allied health findings did not emphasise the opportunity for hands on learning or following patients from the community to hospital, moreover there were some reports of steep learning curves and heavy caseloads whilst transitioning to new roles. This suggests that allied health professionals may expect to have opportunities for hands on learning early in their career and that following up on patients’ needs in hospital or the community was not a significantly unusual experience for them. It was also evident that both groups experienced high expectations on their workload and competency development early in their career. A recent review also reported while broad experiences often draw early career health professionals in, the heavy, complex workloads often impact negatively on retention [16].

A large number of medicine findings related to post graduate training. In some instances early career doctors reported positive opportunities for learning and specialisation in rural areas. In other instances, they reported challenges accessing required training, including the employer not prioritising the time needed for training, geographical and technological challenges and limited options for specialisation. A recent review by Holloway [11] identified similar training challenges for rural doctors and also limitations with backfill to enable doctors to leave town to access training. Some allied health professionals experienced good access to professional development activities with adequate funding and support to attend while others reported heavy workloads, inadequate funding and geographical challenges as having a negative impact on their access to training. This is consistent with a recent review exploring priorities for allied health retention which identified the need to provide allied health professionals with access to the right training and support in order to meet their communities needs [61]. Medicine findings discussed training programs having a link to the specialisation of skills and subsequent career advancement opportunities. Early career doctors reported needing to choose a speciality area and this was a complex process for some while others reported limited opportunities for specialisation outside of general practice and anaesthetics. The development of specialties with associated career paths was not a focus of allied health papers, some findings described good opportunities for leadership in rural areas early in their career, while others reported the opposite with a lack of senior roles impacting on career advancement. A review by Roots and Li [62] also found career advancement limitations in rural areas for occupational therapists and physiotherapist was a challenge that negatively impacted job satisfaction and retention. A rural generalist pathway for early career allied health professionals was introduced in Australia 2014 [63] in an attempt to give early career clinicians the opportunity to develop specialised rural practice skills, leadership and service development skills. At the time of this review, the rural generalist pathway does not appear to lead to a recognised career path or endorsement of specialty status. Experiences relating to the allied health rural generalist pathway did not feature in the papers included in this review.

Allied health and medicine findings suggest the location of family, partner or friends is an important factor in clinicians overall experience of working in a rural area which is widely recognised in the literature [5, 11, 16]. In this review a range of medical findings related to doctors feeling supported when their family or partner were co-located with them or when they were close enough to visit on weekends and having a negative experience when their significant others were away. Allied health professionals reported being away from family or a partner was a significant challenge and a couple of papers reported having a partner locally was a reason to stay. Early career doctors were particularly concerned with the needs of their partner and children, which was extensively reported. Allied health professionals reported they would leave a rural area in the future to be closer to their family, but interestingly did not report bringing their partner or family with them to the rural area. Allied health professionals undertake a 4 or 5 year degree to qualify [17] whereas doctors are at university for longer. Potentially allied health professionals are moving to rural areas at a younger age, in contrast to doctors, and may not have yet established a family.

Allied health professionals reported challenges finding accommodation in rural areas with some reports of clinicians sharing a house to manage the challenge. Some allied health professionals were commuting long distances each day to get to work or staying in the rural area during the week and going home on the weekends. Challenges of sourcing accommodation were not reported in the medicine papers except for one findings that identified accommodation was provided by the employer but was not satisfactory.

Consistent with previous studies [5, 64] feeling welcomed and connected to the community was reported to be important in this review. Some allied health professionals discussed feeling welcome and included in the local community, while others reported it was challenging to get to know people, to feel involved and to find out what activities were available within the community. Conversely a range of medicine papers that discussed community integration reported doctors were welcomed into the local community, involved in activities outside of work, and enjoyed the rural lifestyle. A small number of studies reported community integration and social isolation as being challenging for early career doctors. Allied health professionals also reported challenges with personal and professional boundaries in small towns but the doctors generally reported being well known in rural areas was a positive aspect of rural work.

### Implications

The findings of this review have outlined the similarities and differences in experiences of early career allied health professionals and doctors. These findings have implications for rural and remote health services, policy makers and future researchers;

- A structured career path for early career doctors in areas other than general practice and anaesthetics similar to opportunities available in metropolitan areas might encourage more doctors to train and work in rural areas.
- Giving allied health professionals career advancement opportunities in rural areas through specialist or generalist training that result in recognition of expertise, and a pathway of rural career progression might enable clinicians to plan a rural career.
- Local supervision and prioritised access to post graduate training for junior doctors will provide a more positive experience.
- Allied health professionals need access to discipline specific supervision in order for them to develop confidence and competence.
- Acknowledging and addressing personal factors including the location and needs of a partner or family, integration into the community and consideration of personal and professional boundaries may result in a more positive experience for all health professionals.

### Strengths and limitations

It is important to consider the strengths and limitations of this review. This systematic review includes searches across eight databases, two reviewers screened titles and abstracts and full text articles. All four reviewers were involved in extraction of included studies. A meta-analysis was conducted to identify findings and relevant illustrations, categories and synthesised findings exploring the experience of working in rural and remote areas as an early career doctor or health professional. A limitation of this review included the challenge of identifying papers that focused on early career professionals. A range of studies were excluded because it was not clear how experienced the health professionals were that were investigated. Allied health professionals generally stay in rural or remote areas for up to three years [3] and doctors for seven years [14] so it may have been possible to include more studies if the authors had stated the years of experience of their participants. Furthermore, some studies were excluded because the experiences of early career health professionals were reported through the perspectives of managers, employers or students rather than the early career clinician themselves.

While the appraisals indicated the quality of the articles was moderate to high, one study did not outline their methods clearly and so the appraisal was rated low. The reviewers noted that although the methods were not clearly outlined this is not necessarily a reflection of the quality of the research methods. The findings from this article were considered in relation to the other studies and were found to be consistent. Limitations with studies related to identifying the perspectives and potential biases of the researchers. Caution should therefore be observed when considering the findings of this review.

Other limitations are that only papers published in English, from high income countries, and with a defined definition of allied health were included. This ensured both the comparability in the context of the rural and remote experiences, enabling the meta-synthesis of results; and facilitated the development of policy and workforce recommendations for similar contexts. This review may have therefore been subjected to publication bias.

This research has considered experiences once clinicians are working in rural areas. Future research could focus more closely on the impact of personal and organisational factors on recruitment for early career health professionals to identify strategies to attract more people to work in rural areas.

## Conclusion

Early career allied health professionals and doctors experience a range of similarities and differences working in rural areas. There is a complex interplay of factors needed to support rural health professionals career path and retention. Considering the factors as a whole, it is apparent that there is a need for a review of workforce structures in rural and remote areas to facilitate retention of both the allied health and doctor workforce. Common areas for consideration were: access to structured training programs enabling the development of recognised generalist or specialist career paths regardless of health profession; availability of a range of hands on learning opportunities in their jobs; consistent, high quality supervision; and a whole of community approach to workforce to facilitate opportunities for both family members, and the health professional to be part of the rural and remote community.

## Supplementary Information

**Additional file 1.**

**Additional file 2.**

## Data Availability

The datasets analysed during the current study are not publicly available due some of the raw data being accessed under licence but are available from the corresponding author on reasonable request.

## Abbreviations

JBI: Joanna Briggs Institute
PRISMA: Preferred Reporting Items for Systematic Review and Meta-Analyses
