# Supplementary material for: Experiences of working as early career allied health professionals and doctors in rural and remote environments: a qualitative systematic review
Source: BMC Health Serv Res. 2022 Jul 26;22:951. doi: 10.1186/s12913-022-08261-2 (PMC9327222; doi:10.1186/s12913-022-08261-2)
Supplement: Supplementary file 1 — Additional file 1. [file 12913_2022_8261_MOESM1_ESM.docx]

### Credibility of findings by study

| **Citation** | **Credible** | **Unequivocal** |
| --- | --- | --- |
| Bayley et al. | 1 | 8 |
| Bonney et al | 1 | 7 |
| Brown et al |  | 6 |
| Campbell et al | 3 | 4 |
| Cleland et al | 3 | 12 |
| Cosgrave et al | 1 | 24 |
| Cuesta-Briand et (b) | 4 | 11 |
| Cuesta-Briand et al (a) | 1 | 7 |
| Devine | 14 | 5 |
| Devine et al | 5 | 1 |
| Doyle et al | 1 | 4 |
| Edwards et al |  | 4 |
| Elliott et al |  | 21 |
| Gill et al |  | 14 |
| Iedema et al. |  | 4 |
| Isaacs et al. |  | 16 |
| Keane et al.. |  | 4 |
| Lee et al. | 5 | 13 |
| Malau-Aduli et al. | 1 | 8 |
| Martin et al. |  | 14 |
| McKillop et al.. |  | 7 |
| Mugford et al. | 8 | 2 |
| Myhre et al. |  | 6 |
| Pandit et al. | 2 | 4 |
| Peel et al. |  | 3 |
| Smith DM. 2005. | 7 | 3 |
| Steenbergen et al. | 13 |  |
| Thackrah et al. | 9 | 8 |
| Walters et al. | 7 | 4 |
| Wearne et al. | 10 | 5 |
