# Supplementary material for: Experiences of working as early career allied health professionals and doctors in rural and remote environments: a qualitative systematic review
Source: BMC Health Serv Res. 2022 Jul 26;22:951. doi: 10.1186/s12913-022-08261-2 (PMC9327222; doi:10.1186/s12913-022-08261-2)
Supplement: Supplementary file 2 — Additional file 2. [file 12913_2022_8261_MOESM2_ESM.docx]

### Critical Appraisal Results

| **Citation** | **Q1** | **Q2*** | **Q3*** | **Q4*** | **Q5** | **Q6*** | **Q7*** | **Q8** | **Q9** | **Q10** |
| --- | --- | --- | --- | --- | --- | --- | --- | --- | --- | --- |
| Bayley et al. | Y | Y | Y | Y | Y | N | N | Y | Y | Y |
| Bonney et al | U | Y | Y | Y | Y | N | N | Y | Y | Y |
| Brown et al | Y | Y | Y | Y | Y | N | N | Y | Y | Y |
| Campbell et al | Y | Y | Y | Y | Y | N | N | Y | Y | Y |
| Cleland et al | Y | Y | Y | Y | Y | N | N | Y | Y | Y |
| Cosgrave et al | Y | Y | Y | Y | Y | N | N | Y | Y | Y |
| Cuesta-Briand et al Understanding factors… | Y | Y | Y | Y | Y | N | N | Y | Y | Y |
| Cuesta-Briand et al Extending conceptual framework | Y | Y | Y | Y | Y | N | N | Y | Y | Y |
| Devine | Y | Y | Y | Y | Y | N | Y | Y | Y | Y |
| Devine et al | N | Y | Y | Y | Y | N | N | Y | Y | Y |
| Doyle et al | U | Y | Y | Y | Y | N | N | Y | Y | Y |
| Edwards et al | Y | Y | Y | Y | Y | N | N | Y | Y | Y |
| Elliott et al | Y | Y | Y | Y | Y | Y | Y | Y | Y | Y |
| Gill et al | Y | Y | Y | Y | Y | N | N | Y | Y | Y |
| Iedema et al. | U | U | Y | U | U | N | N | Y | Y | Y |
| Isaacs et al. | U | Y | Y | Y | Y | N | N | Y | Y | Y |
| Keane et al.. | Y | Y | Y | Y | Y | N | Y | Y | Y | Y |
| Lee et al. | Y | Y | Y | Y | Y | N | Y | Y | Y | Y |
| Malau-Aduli et al. | Y | Y | Y | Y | Y | N | Y | Y | Y | Y |
| Martin et al. | Y | Y | Y | Y | Y | N | Y | Y | Y | Y |
| McKillop et al.. | Y | Y | Y | Y | Y | N | N | Y | Y | Y |
| Mugford et al. | Y | Y | Y | Y | Y | N | N | Y | N | Y |
| Myhre et al. | Y | Y | Y | Y | Y | N | N | Y | Y | Y |
| Pandit et al. | Y | Y | Y | Y | Y | N | Y | Y | Y | Y |
| Peel et al. | Y | Y | Y | Y | Y | Y | Y | Y | Y | Y |
| Smith DM. 2005. | Y | Y | Y | Y | Y | N | Y | Y | N | Y |
| Steenbergen et al. | Y | Y | Y | Y | Y | N | N | Y | Y | Y |
| Thackrah et al. | Y | Y | Y | Y | Y | N | Y | Y | Y | Y |
| Walters et al. | Y | Y | Y | Y | Y | Y | Y | Y | Y | Y |
| Wearne et al. | Y | Y | Y | Y | Y | Y | Y | Y | Y | Y |
|  | 83.3% | 96.7% | 100.0% | 96.7% | 96.7% | 13.3% | 40% | 100.0% | 93.3% | 100.0% |
